# Does Preterm Prolonged Rupture of Membranes Increase the Risk of Needing Invasive Respiratory Support? A Retrospective Single-Centre Study

**DOI:** 10.3390/children11070823

**Published:** 2024-07-05

**Authors:** Eleanor Jeffreys, Ravindra Bhat, Anne Greenough, Theodore Dassios

**Affiliations:** 1 Department of Women and Children’s Health, School of Life Course Sciences, Faculty of Life Sciences and Medicine, King’s College London, London SE5 9RS, UK; eleanor.jeffreys@nhs.net (E.J.); anne.greenough@kcl.ac.uk (A.G.); 2 Neonatal Intensive Care Centre, King’s College Hospital NHS Foundation Trust, London SE5 9RS, UK; ravindra.bhat@nhs.net

**Keywords:** premature, prolonged rupture of membranes, invasive ventilation

## Abstract

(1) Background: Preterm premature rupture of membranes (PPROM) has been associated with increased perinatal morbidity, but the effect of PPROM on respiratory disease has not been previously quantified. We hypothesised that PPROM would be associated with a higher incidence of invasive ventilation. (2) Methods: A retrospective cohort study at the Neonatal Unit at King’s College Hospital NHS Foundation Trust, London, UK, was conducted on infants born before 37 weeks of gestation. PPROM was defined as the rupture of membranes for >48 h. (3) Results: We reviewed 1901 infants (434 with PPROM) with a median (IQR) gestational age of 32.4 (28.7–35.0) weeks. The median (IQR) duration of rupture of membranes in the infants with PPROM was 129 (78–293) h. The incidence of invasive ventilation was 56% in the infants with PPROM and 46% in the infants without PPROM (*p* < 0.001). Following regression analysis, PPROM was significantly related to a higher incidence of invasive ventilation (odds ratio: 1.48; 95% CI: 1.13–1.92, adjusted *p* = 0.004) after adjusting for birth weight [odds ratio = 0.34; 95% CI: 0.33–0.43, adjusted *p* < 0.001], Apgar score at 10 min [odds ratio =0.61; 95% CI: 0.56–0.66, adjusted *p* < 0.001] and antenatal corticosteroid use (adjusted *p* = 0.939). (4) Conclusions: PPROM was associated with a 1.48-fold higher risk of needing invasive ventilation.

## 1. Introduction

Preterm premature rupture of membranes (PPROM) is defined as spontaneous membrane rupture occurring prior to the onset of labour, before 37 weeks of gestation. The latency period (the time between membrane rupture and delivery) varies, with 50% of women experiencing PPROM giving birth within 24–48 h after the rupture and 70–90% giving birth within 7 days [1]. PPROM occurs in 3% of pregnancies and is associated with 30–40% of all preterm births [2]. PPROM is an important cause of perinatal morbidity and mortality, with respiratory distress syndrome (RDS) being the most common serious neonatal complication at any gestational age [2]. Neonatal outcomes following PPROM are affected by preterm delivery, the degree of the inflammatory response and the presence of pulmonary hypoplasia resulting from the reduced amniotic fluid at the very early stages of gestation (less than 25 weeks) prior to full lung development and growth [3,4]. It has been shown that neonatal sepsis is twice as common following PPROM compared with preterm birth with intact membranes [5]. Both pulmonary hypoplasia [6] and perinatal infection [7] have been shown to increase the need for respiratory support in premature infants, and PPROM is therefore believed to be associated with increased respiratory morbidity and an increased need for intubation and ventilation.

Currently, there are few studies that have looked at the effect of PPROM on respiratory morbidity, and the effect of PPROM on the incidence of invasive ventilation has not been previously quantified. We used the need for invasive ventilation as an objective proxy for respiratory disease, since radiological and laboratory findings might vary and there is wide variation regarding the types of non-invasive respiratory support used. 

We hypothesised that PPROM would be associated with a higher incidence of invasive ventilation and that the duration of ventilation would be significantly related to the duration of rupture of membranes. Our aim was to test those hypotheses.

## 2. Materials and Methods

A retrospective cohort study of all admissions to the Neonatal Unit at King’s College Hospital NHS Foundation Trust, London, UK (KCH), of infants born before 37 completed weeks of gestation between 1 January 2012 and 1 January 2023 (11 years) was undertaken. The starting year of 2012 was selected for the purpose of data entry consistency and to reflect current neonatal practice. KCH has a tertiary neonatal unit with approximately 6000 deliveries per year and serves a diverse community of over 1,000,000 in South-East London by providing all levels of neonatal care (intensive care, high-dependency and special care). As the neonatal unit at KCH is a regional surgical and neurosurgical referral centre, the analysis excluded major congenital surgical diagnoses such as congenital diaphragmatic hernia, gastroschisis, exomphalos, oesophageal atresia, bowel atresia, Hirschsprung’s disease, congenital pulmonary airway malformation, congenital hydrocephalus, spina bifida and Trisomy 21. Data were extracted from the BadgerNet Neonatal Electronic Patient Database (Clevermed, Edinburgh, UK). 

The following data were collected: maternal age (years); at least one complete course of antenatal steroids (betamethasone) (yes/no) [8]; duration of rupture of membranes (h), prolonged premature rupture of membranes, defined as rupture of membranes for more than 48 h (yes/no) [8]; sex (male/female); gestational age (weeks); birth weight (kg), Apgar score at 10 min; admission temperature (°C); invasive mechanical ventilation (yes/no), duration of invasive ventilation (days). Mortality was defined as death before discharge from neonatal care. Infants were intubated if they had respiratory acidosis (pH < 7.2), apnoea or significant work of breathing on non-invasive support and were ventilated on synchronised pressure-limited or volume-targeted ventilation [9]. High-frequency oscillation was used as a rescue treatment for infants requiring a mean airway pressure above 18–20 cm H_2_O. Intubated preterm infants with an oxygen requirement exceeding 30% received intratracheal surfactant [9]. The study was registered with the Clinical Governance Department of KCH. The Health Research Authority Toolkit of the National Health System, United Kingdom, confirmed that the study did not need regulatory approval by a research ethics committee.

### Statistical Analysis

Continuous data were tested for normality with the Kolmogorov–Smirnov test, found to be non-normally distributed and therefore presented as a median and interquartile range (IQR). The primary analysis aimed to determine if there was a statistically significant difference in the incidence of invasive ventilation in infants with PPROM versus the ones without PPROM and was undertaken using the x^2^ test. The incidence of caesarean section, antenatal steroids and male sex was compared between ventilated and non-ventilated infants using the x^2^ test. The gestational age, birth weight, Apgar score at 10 min and admission temperature were compared in ventilated and non-ventilated infants using the Mann–Whitney U non-parametric test. The relation of the duration of rupture of membranes with the duration of ventilation was examined using Spearman’s Rho non-parametric correlation analysis after excluding infants who died. The independent contribution of PPROM to the incidence of ventilation was examined using binary regression analysis after adjusting for parameters that were significantly different between ventilated and non-ventilated infants (*p* < 0.10) at a univariate level. Multi-collinearity among the independent variables in the regression analysis was assessed by examining a correlation matrix for the independent variables. 

Statistical analysis was performed using SPSS software, version 27.0 (SPSS Inc., Chicago, IL, USA).

## 3. Results

**Figure 1:**
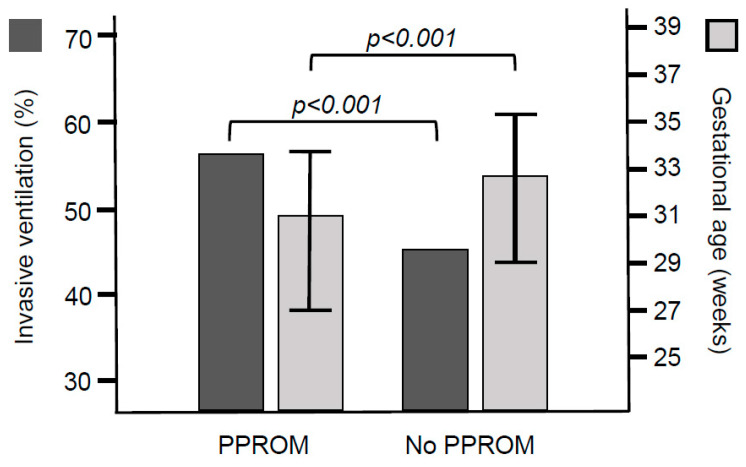
Incidence of invasive ventilation and median (interquartile range) values of the gestational age in infants with and without PPROM.

In the study period, 3646 infants born at less than 37 weeks of gestation were admitted to the Neonatal Unit at KCH, of whom 132 had a major congenital surgical diagnosis. A further 1613 infants were excluded for missing data for the exact duration of rupture of membranes (44%). The final analysed cohort included 1901 infants, of whom 434 (23%) had PPROM and 1467 (77%) did not. The median (IQR) duration of ROM in the infants with PPROM was 129 (78–293) h. The infants with PPROM more frequently had antenatal steroids and were of a lower gestational age (Figure 1) and birth weight compared with infants without PPROM (Table 1). The infants with PPROM had a lower Apgar score at 10 min and a higher admission temperature compared with infants without PPROM (Table 1). A great majority of the infants who died, died in the first 72 h of life, due to severe RDS and extreme prematurity. 

The incidence of invasive ventilation was 56% (245 of 434) in the infants with PPROM and 46% (673 of 1467) in the infants without PPROM (*p* < 0.001, Table 1, Figure 1). The incidence of male sex and maternal age was not different in ventilated versus non-ventilated infants. Ventilated infants more frequently had antenatal corticosteroids (806 of 913 infants, 88%) compared with non ventilated infants (711 of 977 infants, 73%, *p* < 0.001). The median (IQR) admission temperature was not significantly different between ventilated [3.8 (36.5–37.0) °C] and non-ventilated infants [36.8 (36.5–37.1) °C, *p* = 0.559]. The median (IQR) gestational age [29.4 (26.0–32.9) weeks] and birth weight [1.24 (0.81–1.78) kg] were lower in the ventilated infants compared with the gestational age [34.1 (32.0–36.0) weeks] and birth weight [1.95 (1.55–2.41) kg] in non-ventilated infants [*p* < 0.001 for both]. The median (IQR) Apgar score at 10 min was lower in ventilated infants [9 (8–10)] compared with that in non-ventilated infants [10 (9–10), *p* < 0.001].

Following binary regression analysis with the incidence of ventilation as the outcome parameter, PPROM was significantly related to a higher incidence of invasive ventilation (odds ratio: 1.48; 95% CI: 1.13–1.92, adjusted *p* = 0.004) after adjusting for birth weight [odds ratio = 0.34; 95% CI: 0.33–0.43, adjusted *p* < 0.001], Apgar score at 10 min [odds ratio = 0.61; 95% CI: 0.56–0.66, adjusted *p* < 0.001] and antenatal corticosteroids (adjusted *p* = 0.939). Gestational age was not included in the model due to its collinearity with birth weight.

The median (IQR) duration of invasive ventilation was not significantly different in ventilated infants with PPROM [3 (1–14) days] compared with that in ventilated infants without PPROM [3 (1–14) days, *p* = 0.667] and was not significantly related to the duration of rupture of membranes (rho = −0.032, *p* = 0.336).

## 4. Discussion

We demonstrated that PPROM was significantly associated with a higher incidence of invasive ventilation in preterm infants but the duration of ventilation was not associated with the duration of rupture of membranes. Additionally, infants born following PPROM were more likely to have received antenatal steroids, and had a lower gestational age, a lower birth weight and higher mortality. 

These data agree with the findings by Muller et al. [10], who found significant correlations between the duration of PPROM and respiratory distress syndrome and need for surfactant administration. Interestingly, they found no correlation between the duration of PPROM and the incidence of bronchopulmonary dysplasia (BPD). More recently, Paulsen et al. [11] showed that 76.1% of infants born following PPROM were observed to have RDS. They showed a significant mortality rate of 28.1%, which despite being higher than the rate we found in this study, did concur with our findings of higher mortality in preterm infants following PPROM. They went on to show a significant association between a diagnosis of RDS and the later development of BPD. 

Conversely, a large German study [12] on 4120 infants found no increase in the rate of surfactant administration (*p* = 0.92), no increase in invasive ventilation (*p* = 0.20) and no increase in the duration of ventilation (*p* = 0.40) or duration of oxygen supplementation (*p* = 0.21) in infants who had been born following PPROM. Following logistic regression, they did find that infants born following PPROM had an OR of 1.25 (CI 1.02–1.55) of developing BPD. Of note is that they did not comment on the latency period between PPROM and birth. Their gestational ages (mean) were 28.1 weeks (no PPROM) and 27.7 weeks (PROM), and their birth weights (mean) were 1005 g (no PPROM) and 1045 g (PPROM), which are lower than those of the cohort in our study. This could indicate a sicker population, leading to greater respiratory morbidity in both groups, perhaps masking the effect of PPROM on early respiratory morbidity. 

In this study, we did not investigate the effect of PPROM on the development of BPD. This was due to the inclusion of all babies <37 weeks gestation, meaning that a large proportion of the cohort were late preterm infants. Since BPD is much less frequent in the late preterm population, the effect of PPROM on BPD in our cohort would have been limited by the large number of infants in our study.

The higher incidence of antenatal corticosteroids in our study in infants with PPROM seems surprising at first, but is likely a reflection of the slightly different ranges of gestational age between the groups of infants with and without PPROM. As the recommendation for antenatal steroids during our study was to give them to mothers of infants below 35 weeks of gestation, the larger median gestational age of infants without PPROM in our study might explain why they received less antenatal steroids compared with their more premature counterparts with PPROM [8]. 

Our results showed that PPROM significantly influenced the need for invasive ventilation; however, it did not affect the duration of invasive ventilation. This implies that in our population and for our given definition of PPROM with a cut-off of 48 h, the main mechanism by which PPROM increased respiratory morbidity was through ascending infection rather than lung hypoplasia. A longer latency of PPROM, which would be associated with pulmonary hypoplasia, was not associated with a longer period of ventilation, as would be expected in the case of hypoplastic lungs. In light of this, it is important to consider the clinical applicability of our findings. PPROM was associated with a 1.5-fold higher risk of intubation and ventilation in preterm infants. However, by prolonging the latency and allowing antenatal steroids to act, the lungs were given additional time to mature. The balance between lung maturation and the risk of increasing infection needs to be considered and likely needs to be assessed on an individual basis. 

It was not possible from our data to accurately measure and record chorioamnionitis. This can be a difficult diagnosis; the condition is largely underdiagnosed clinically, and often not proven to be present due to difficulty in achieving a histological examination of the placenta. Our results, however, suggest that PPROM could be associated with an increased incidence of maternal/neonatal infection, and concur with recent evidence that chorioamnionitis could influence the respiratory drive, leading to a need for additional respiratory support at birth [7]. The same applies for neonatal sepsis, which often proves to be an evasive diagnosis with a high percentage of culture-negative events. 

The strengths of this paper are the large sample size and the time frame in which the data were collected. Possible limitations include the fact that our study was conducted retrospectively at a single centre with a significant proportion of subjects having incomplete data and being eventually excluded. We were not able to differentiate between RDS and transient tachypnoea of the newborn as the primary reason for intubation and ventilation. These diagnoses are not always clearly separated in clinical practice. Additionally, it is not possible to clearly delineate the contribution of infection to respiratory morbidity, as some element of infection might exacerbate respiratory disease or directly cause pulmonary infection. 

In conclusion, our study demonstrated that PPROM was associated with a 1.5-fold higher risk of the need for intubation and ventilation in preterm infants.

## Figures and Tables

**Table 1:** Characteristics and outcomes of the included infants according to whether they had PPROM or not. Data are presented as median (IQR) or *N* (%).

	PPROM *N* = 434	No PPROM *N* = 1467	*p* Value
Maternal age (years)	33 (28–36)	33 (28–36)	0.250
Antenatal steroids	390 (90)	1127 (77)	<0.001
Duration of ROM (h)	129 (78–293)	0 (0–8)	<0.001
Male sex	249 (57)	818 (56)	0.753
Gestational age (weeks)	31.0 (27.1–33.7)	32.8 (29.0–35.3)	<0.001
Birth weight (kg)	1.51 (0.94–2.04)	1.70 (1.08–2.26)	<0.001
Birth weight z-score	−0.33 (−0.73 to 0.18)	−0.40 (−1.16 to 0.19)	0.001
Apgar score at 10 min	9 (8–10)	10 (9–10)	0.009
Admission temperature (°C)	36.8 (36.5–37.1)	36.7 (36.5–37.0)	0.009
Invasive ventilation	245 (56)	673 (46)	<0.001
Died	23 (5.3)	39 (2.7)	0.007

PPROM: prolonged preterm rupture of membranes.

## Data Availability

The raw data supporting the conclusions of this article will be made available by the authors on request.
